# Case of Equine Rabies

**Published:** 1896-03

**Authors:** B. M. Underhill

**Affiliations:** Media, Pa.


### CASE OF EQUINE RABIES.

By B. M. UNDERHILL, V.M.D.,
MEDIA, PA.

PATIENT, chestnut gelding, nine years of age, used as family driver. Sunday afternoon acted strangely, restless, but symptoms slight and not regarded as important.

Monday A.M., symptoms slightly intensified. Was put under saddle for short trip; would not keep to trot (his natural gait), but persisted in a fretful gallop, a disposition and gait that his rider never before noticed; ordinarily he was said to be very docile, and used and made a pet of by all members of the family.

In the afternoon he was harnessed to a buggy and driven upon the streets. The driver noticed such an unusual fretfulness that he called the attention of a friend to the condition, and the latter walked around in front of the horse, when the animal, with jaws wide apart, made a lunge at him and attempted to bite. Shortly afterward he made a similar attempt upon a boy who ventured in reach.

He was taken to the stable and Dr. Young, of Media, called. Dr. Young regarded him with suspicion, but led him to a box-stall at his own stable, where he left an attendant to watch him during the night. While being led by Dr. Young he made two attempts to bite him, but was beaten off with a stick.

The following morning Dr. Brodhead, of Media, and myself were called in consultation, and, from what history we could get and symptoms observed, we pronounced it undoubtedly a case of rabies. At this time the animal was repeatedly stamping and kicking violently and spasmodically with one foot.

He would rush at anyone moving toward him, with mouth wide open, and thrust his muzzle as far as possible between the iron bars of his stall in a frantic effort to get at the object of his attack. Failing in this, he would seize the woodwork and tear pieces from it, on one or two occasions breaking his incisors.

It being necessary for Dr. Young to leave, the case was turned over to me, and after giving a number of the veterinary students of the University of Pennsylvania an opportunity to see him, I directed that he be shot by an expert marksman whom I had on hand in case the horse should break from his stall, which occurrence at certain periods did not seem unlikely.

Before his destruction the horse had become quite violent, kept up a constant champing of his jaws, and appeared much irritated at the crowd of a hundred or more people who had collected about the stable. The expression at no time was what is commonly called wild, but was rather one of anticipation, continually on the alert; neither pupil was dilated. He would swallow small quantities of water, and when hay was held out to him he would rush to attack it, but would take the hay and chew it normally. The constant movement of the jaws had caused a frothy saliva to collect about the lips. His attacks were always with malice and frantic attempt to do injury. During an attack he would obey the command of his attendant and jump back in apparent fear.

I could get very little history from the owner that would shed any light as to how the animal became inoculated, but I finally got it from a neighbor that three months ago the owner had a Newfoundland dog which acted strangely, hid under the barn and would not come out; he finally succeeded in getting the dog out and shut him in the stable at night with this horse; next morning found he had escaped from the stable and was never heard of afterward. I also found upon further investigation that about a month previously to this a strange dog had taken refuge under a porch on the premises, and was there shot, and was thought by some then present to be rabid. Fortunately, this horse has bitten neither man nor beast, and there will be no excursions to the Pasteur Laboratory.

We have so little chance to observe rabies in the horse that Dr. W. L. Rhoads, who was present, and myself thought the details of this case would perhaps be of interest to others of the veterinary profession.

